# Use it or lose it: a qualitative study of the maintenance of physical activity in older adults

**DOI:** 10.1186/s12877-019-1366-x

**Published:** 2019-12-12

**Authors:** Asiya Maula, Natasher LaFond, Elizabeth Orton, Steve Iliffe, Sarah Audsley, Kavita Vedhara, Denise Kendrick

**Affiliations:** 1 School of Medicine, University of Nottingham, Floor 14 Room 1401, Tower building, University Park, Nottingham, NG7 2RD UK; 2 Research department of Primary Care & Population Health, University College London, Gower Street, WC1E 6BT London, UK

**Keywords:** Physical activity, Maintenance, Older adults, Qualitative study, Community exercise programmes

## Abstract

**Background:**

Lack of physical activity (PA) is a recognised global public health problem, which is increasing in prevalence with a detrimental impact on the pattern of disease worldwide. In the UK, older adults comprise the most sedentary group, with only 57% of males and 52% of females aged 65–74 years and 43% of males and 21% of females aged 75–84 years meeting PA recommendations.

PA confers multiple health benefits including increased stamina, muscle, bone and joint strength, increased independence and reduced risk of falls in old age. Despite benefits experienced during time-limited PA programmes, increased PA is not always continued. This study aimed to provide a better understanding of PA maintenance behaviours in older people.

**Methods:**

Face to face semi-structured interviews were conducted with adults who completed one of two strength and balance exercise programmes as part of the ProAct65+ trial: group (FaME) and home based (OTAGO) exercises. Five GP practices in Nottingham and Derby were recruited and invited people aged 65 years and older who met eligibility criteria. Interviews were conducted in participants’ homes. Interviews explored PA levels pre and post intervention, perceived health benefits, facilitators, barriers and use of technology for PA maintenance. The interviews were transcribed verbatim and analysed using framework analysis and the software NVivo10.

**Results:**

Fifteen participants from each intervention group were interviewed. The FaME group consisted of 10 females and 5 males, age range of 70–88 years. The OTAGO group consisted of 12 females and 3 males aged 72–95 years. Important themes identified were physical, social, psychological and environmental facilitators and barriers. These included increased physical autonomy, enjoyment, positive evaluation of the activity and physical benefits, importance of social interaction, positive feedback, development of behaviour considered normal or habitual, motivation and self-efficacy. Some participants used technologies not included in the original interventions, like pedometers and smart phones to motivate themselves.

**Conclusions:**

A range of modifiable factors influence continued participation in PA at the end of exercise programmes. The findings from this study will inform the commissioning and quality improvement of future PA programmes and development of an intervention to enhance continuation of PA after exercise interventions in older adults.

## Background

Physical inactivity is ‘the non-achievement of physical activity guidelines’ [1] defined by Public Health England as ‘engaging in less than 30 minutes of physical activity per week’ [2]. It is the fourth leading risk factor for global mortality [3], responsible for 9% of premature deaths worldwide in 2008 [4]. Inactivity increases susceptibility to chronic conditions (e.g. type II diabetes, osteoporosis, cancer, cardiovascular disease, falls) which are major causes of morbidity, mortality and health resource use worldwide [5–10]. It is estimated that a reduction in inactivity would lessen the burden of the leading non communicable diseases (which include coronary heart disease, type 2 diabetes, breast and colon cancers) by between 6 and 10% worldwide and improve life expectancy [4].

The population of older adults is increasing alongside the prevalence of chronic diseases. In the UK, between 2001 and 2011, the population of adults aged 65 years and over increased by approximately 1 million [11]. Worldwide the number of adults aged 60 years and over is estimated to increase 56% between 2015 and 2030 [12]. In the UK, older adults comprise the most sedentary group. Current recommendations for physical activity (PA) amongst older adults are 150 min of moderate to vigorous aerobic exercise and 2 strength and balance exercise sessions per week [13]. However, only 57% of males and 52% of females aged 65–74 years and 43% of males and 21% of females aged 75–84 years self-report activities that meet PA recommendations [14].

Regular PA in older people is associated with increased functional independence, stamina (the ability to sustain prolonged physical effort) [15], muscle strength, bone and joint health and improvements in blood pressure. Other benefits include reduced risk of falls and fractures, cardiovascular events, pain and disability from arthritis, symptoms of depression and anxiety and maintenance of cognitive function [6, 16–18]. Many of the positive effects from endurance and resistance type exercise decline within 2 weeks if activity levels are markedly reduced and disappear over 2–8 months if PA levels are not recommenced [18], highlighting the importance of PA maintenance. Whilst there is evidence that promoting physical activity for older people can be effective [19–22], evidence relating to which intervention components help to maintain increases in PA after taking part in structured community-based exercise programmes is much sparser, with only a small number of studies reporting effective PA maintenance [23–33]. Over the last 30 years, numerous theories of physical activity behavioural change have been proposed [34]. We did not choose to focus on one specific behaviour change theory in the design and conduct of our study, instead we use a range of behaviour change theories to interpret our findings.

This study aimed to identify the facilitators and barriers to the maintenance of PA amongst older people after taking part in a three-arm trial of time-limited community-based exercise programmes (Falls Management Exercise programme (FaME) or home based exercise Programme (OTAGO) or usual care) in the ProAct65+ multicentre randomised controlled trial [35]. The ProAct65+ trial recruited community dwelling adults aged ≥65 years, who were independently mobile (with or without a walking aid) and physically able to take part in group exercise. The OTAGO and FaME programmes were both designed for use in community settings, specifically for people aged 65 and over. As well as being designed to reduce falls, both are based on the components of fitness and principles of programming for all older adults (i.e. warm up, mobility, stretches, strength and balance, endurance and a cool-down) and have all the elements of training appropriate for that age group. Exercises are tailored to the individual’s ability and health need. FaME includes weekly exercise classes, and both FaME and OTAGO include home exercises. The primary outcome of the ProAct65+ trial was self-reported physical activity, and falls were a secondary outcome. The trial demonstrated a significant increase in the proportion of older people undertaking at least 150 min of moderate to vigorous physical activity per week and a significant reduction in the incidence of falls in the FaME arm compared to the control group, 12 months after the intervention. Full details of the trial are available at: www.ncbi.nlm.nih.gov/books/NBK262322/

The findings from this qualitative study can help us understand why falls prevention exercises can increase general physical activity and could inform the design and delivery of future exercise programmes for older adults.

## Methods

The 10 GP practices in Nottingham and Derby which recruited the largest numbers of participants to the ProAct65+ trial were approached to take part in the study, the first five practices who consented to participate were recruited. The ProAct65+ trial included a six-month intervention period, with follow-up 24 months after the end of the intervention. The intervention period for individual trial participants ended between January 2010 and March 2012. Interviews for the qualitative study took place between December 2015 and March 2016, i.e. between 4 and 6 years post intervention. Practices sent out study invitations including a participant information sheet to ProAct 65+ trial participants who were eligible for the current study (eligibility criteria are shown in Table 1) with reminders 3 weeks later to non-responders.

**Table 1:** Inclusion Criteria

Inclusion criteria	
Previous ProAct65+ participant in either the FaME or OTAGO arms of the trial	
Able to provide written informed consent	
65 years old and above	
English speaking	
Study recruitment considered appropriate by GP and would not cause undue distress to participant	
Not diagnosed with a terminal illness	

Maximum variation sampling was used to ensure diversity across the FaME or OTAGO programmes, gender, age, previous falls and fear of falling. Responders meeting sampling criteria were provided with further study information by telephone, with subsequent semi-structured face to face interviews conducted at the participants’ own home. Written informed consent for the interview was obtained face-face at the participants own home prior to the interview. Withdrawal was permitted at any time point during the study, with consent sought to use the data collected thus far.

Interview questions were based on a review of the relevant literature, expert opinion of project team members and advice from patient and public involvement (PPI) representatives, as recommended by INVOLVE where research is conducted ‘with’ or ‘by’ the public [36]. Interviews sought views about the exercise programme, current PA and PA pre-and post ProAct65+ trial participation, perceived facilitators and barriers to PA maintenance, use of technology in keeping physically active and influence from family or friends on PA (See Additional file 1). The first two interviews served as pilot interviews, after which, the question order was modified to improve participant understanding of the questions, no other changes were made. Pilot interview data was included in the analysis. To ensure consistency of interview technique, interviewer shadowing took place where one researcher conducted the interview and the second observed, followed by debriefing. This process was repeated with roles reversed until both interviewers were satisfied with the consistency of their interview technique. Interviews were conducted by one of three researchers (NL, AM, SA). Interviews were fully completed by all participants.

Participants completed a questionnaire at the interview, including questions on FRAT [37] (fall risk assessment tool), physical activity [38], concern about falling [39] and general health [40]. Interviews were recorded on a digital audio recorder and transcribed verbatim. The transcribed data was managed using NVivo10 and analysed using framework analysis [41]. Immersion of the data ensued to allow familiarization [42]. The transcripts were then coded identifying recurrent themes. The analysis framework was developed based on the codes identified. The first researcher coded all transcripts, nineteen were coded by the second researcher ensuring consistency of themes identified with any discrepancies discussed and resolved. In addition, a PPI representative read and coded three transcripts followed by discussion with researchers, this allowed for incorporation of a lay perspective on themes identified.

## Results

### Demographics

There were 122 ProAct65+ participants in the five recruited practices, 99 of whom met eligibility criteria and were invited to participate in the study. Fifty-three (54%) replied expressing interest. Thirty interviews were conducted between December 2015 and March 2016. Each interview lasted up to 1 h 20 min. Thematic saturation was achieved with the number of interviews completed.

Characteristics of study participants are shown in Table 2. Four main themes were identified: physical, psychological, social and environmental with several subthemes within each. Quotes accompanying each section can be found in Table 3.

**Table 2:** Characteristics of study participants

	Exercise group	
	FaME *n* = 15	OTAGO *n* = 15
Participant Characteristics		
Female sex, n (%)	10 (66.7)	12 (80.0)
Mean age, years [SD] Age range, years	76.47 [5.6] 72–88	78.47 [5.7] 70–95
Household, n (%)		
Lives alone	5 (33.3)	6 (40.0)
Lives with other family members	0 (0.0)	2 (13.3)
Married or living as married	10 (66.7)	7 (46.7)
Employment, n (%)		
In part time employment	1 (6.7)	0 (0.0)
Homemaker	0 (0.0)	1 (6.7)
Retired	14 (93.3)	14 (93.3)
Minutes of moderate to vigorous physical activity per week^a^ Mean [SD]	173 [130.5]	177 [148.8]
Range	0–375	0–540
At least 150 min moderate to vigorous physical activity per week, n (%)	7 (46.7)	9 (60.0)
Current level of activity, n (%)		
I do not do any planned physical activity during the week and would find it difficult to start	0 (0.0)	3 (20.0)
I used to exercise regularly each week but have lapsed	2 (13.3)	0 (0.0)
I exercise once in a while but not weekly	0 (0.0)	4 (26.7)
I exercise regularly each week	13 (86.7)	8 (53.3)
Falls risk^b^ Median [IQR]	1.00 [0–1]	1.00 [0–1]
Had a fall in the last 12 months	0 (0)	4 (26.7)
Taking 4 or more medications	7 (46.7)	7 (46.7)
History of Parkinson’s disease or stroke	0 (0)	1 (6.7)
Balance problems	4 (26.7)	1 (6.7)
Unable to rise from chair of knee height	0 (0)	1 (6.7)
Concern about falling^c^ Mean [SD]	1 [1.5]	1.2 [1.3]
General health:^d^		
Excellent	4 (26.7)	3 (20)
Very good	2 (13.3)	1 (6.7)
Good	5 (33.3)	7 (46.7)
Fair	4 (26.7)	4 (26.7)
Poor	0 (0)	0

*SD* standard deviation, *IQR* interquartile range

^a^Community Healthy Activities Model Program for Seniors (CHAMPS) [43]

^b^Falls Risk Assessment Tool (FRAT; range 0–5) [44]

^c^Falls Efficacy Scale - International [39]

^d^12-Item Short Form Health Survey question: In general would you say your health is… [40]

**Table 3:** Illustrative extracted quotes

Themes	Sub themes	Illustrative data extracts
Physical	Physical benefits	‘*I think once you stop exercising you stiffen up and that then takes a lot of coming back if you don’t keep yourself supple and mobile and well you become a cabbage’(ID26 Female 75-79 years OTAGO)*
		*‘I know that it was doing me good because muscles were aching … and I thought this is doing me good, and if you think it is doing you good, you will continue, won’t you?’ (ID2 Female 80-84 years OTAGO)*
		*‘I was more flexible, I do have a bad back which I have had for erm along… decades‘(ID4 Female 70-74 years FAME)*
		*‘Well you know things like getting… you know getting up out of a chair and things like that’ (ID11 Male 70-74 years FAME)*
	Deterioration in physical health	*‘I can’t run anymore, the body is willing but the flesh is weak, the legs won’t go’ (ID12 Female 80-84 years FAME)*
		*‘I think one thing that has, I have thought about for the elderly and the reason why elderly people don’t do exercise is so many of them start going on medication for one thing and another and statins and erm a lot of medication does affect you and makes you feel lethargic and tired and so I think one of the main reasons with the elderly in stopping doing exercises it is tablets' (ID8 Female 75-79 years FAME)*
		*‘I mean I have got arthritis all over the place, in my spine and in my neck and that sort of erm stops me doing anything too energetic’ (ID13 Female 70-74 years FAME)*
		*‘I am having a little problem with my knees so I don’t walk as much as I used to’ (ID 23 Female 80–84 years OTAGO)*
	Health beliefs	*‘It is very difficult, you know people over 55, they’ve started to think they are getting old, and what do I want to do exercise for? Which I can’t… that is not in my mind but I can see this in people that I talk to you know of my age you know, what the hell do I want to do exercise for?’ (ID5 Male 80-84 years FAME)*
		‘*You wonder if it is through doing them that you have got these pains you know’ (ID28 Female 75-79 years OTAGO)*
		*‘Once you stop ….. it becomes more difficult to start up again’ (ID4 Female 70-74 years FAME)*
		*‘Oh well I am well in my 70’s so you know I have a right to sit down and do nothing. I have got a friend like that and everything I do she says oh I bet you were tired weren’t you?’ (ID8 Female 75-79 years FAME)*
	Witnessing deterioration in others	*‘I think seeing how my husband went down so quick, that motivated me more because you can see how quick you do go down’ (ID30 Female 75-79 years OTAGO)*
Social	Social Interaction	*‘as you get older if you are on your own you need company so there is no company is there sat here? You know what I mean?’ (ID28 Female 75-79 years OTAGO)*
		*‘Yes the social side of it yes because we did… You know when you’re retired and not going to work, it is trying to find something else and still meeting people’ (ID1 Male 70-74 years FAME)*
		*‘It was actually one of the people that went to the Pro-Active……they started going to Tai Chi and it was him that told me about this Tai Chi classes so we enrolled in that, we both enrolled on that so it was you know it was somebody we had met at those classes that talked us in to going or told us about this Tai Chi so we thought we would give it a go and erm like I say because we were all beginners’ (ID11 Male 70-74 years FAME)*
	Motivating factors	*‘You know because I had only recently retired as well so it was thinking of some exercises that I could do or something I could do you know to get me out of the house rather than sitting reading and watching television all the while’ (ID11 Male 70-74 years FAME)*
		‘*I wasn’t sure that I would have the commitment to do it at home, you know, life gets in the way’ (ID4 Female 70-74 years FaME)*
		‘*I would have rather have gone to the classes because I think it encourages you more, when you are at home you have got no stimulation’ (ID30 Female 75-79 years OTAGO)*
	Positive feedback from others	*‘Yes because we’re both more or less go to the same type of things and one pushes the other one on shall we say’ (ID27 Male 75-79 years OTAGO)*
		*‘Yes because we work together, if we’re doing anything it is usually done together’ (ID26 Female 75-79 years OTAGO)*
		*‘And I was showing him some of the exercises and it is when hearing him say that would do you good, now that would be good for you...’ (ID2 Female 80-84 years OTAGO)*
	Habit	‘*We don’t mind doing these Pro-Age 65 exercises, as I say we do them every morning without fail, we always find time for that, then we start to get ready to go out or do a days work whatever’* *(ID26 Female 75-79 years OTAGO)*
		‘*you have got to regiment yourself to do these things haven’t you?’ (ID29 Female 75-79 years OTAGO)*
		*‘you get in a routine really and we have such a jolly time that… and I think we all felt that we benefitted hugely, that no it was good’ (ID7 Female 70-74 years FAME)*
		*‘I just carried on doing them because they were asking you how many times you had done them, if one a regular basis with your legs and the weights and all that sort of thing so I just did it and never thinking that this would be coming along now so I did them because they were part of the exercise’ (ID20 Female 75–79 OTAGO)*
	Carer role	*‘Well because you have got to be regimented yourself to do these things haven’t you? You have got to make sure you do them, my husband was alive then so I had a lot to do looking after him you see so it was a bit difficult at times yes’ (ID29 Female 70-74 years OTAGO)*
Psychological	Measurable activity	*‘I would have liked to have carried on doing it because filling those papers in as well every month gave you the incentive to do it’. (ID30 Female 75-79 years OTAGO)*
		‘*you have got to give them a goal and you have got to let them see that they have achieved something’* *(ID24 Male 75-79 years FAME)*
		*‘When there was no record at all required then I just didn’t bother doing it’ (ID1 Female 70-74 years OTAGO)*
	Self-efficacy	*‘I am used to having… doing things, physically, and on top of that exercising regularly in as an individual…………….. so I am quite capable of stringing together a programme that would suit me’ (ID21 Male 70-74 years OTAGO)*
		*‘I was at the home, doing the home, I would have rather have gone to the classes because I think it encourages you more, when you are at home you have got no stimulation more or less when you’re at home but when you are in a class you try and do the same as everybody else don’t you?'*
		*'And it is the company as well when you’re on your own, you have not got the company to encourage you, you know I tended to start and do the exercises but after about half an hour, I got a little bit fed up with it so I left it off and then perhaps went back to it the next day. Whereas if you’re in a class you do it in one go don’t you?’ (ID30 Female 75–79 OTAGO)*
		*‘I suspect that I wouldn’t have done the home exercises as consciously as going to the classes, I need somebody there to motivate me and to push me really and the advice’ (ID7 Female 70-74 years FAME)*
		*'I think it would have been harder to start with you know, to start with. I am usually pretty motivated anyway, if I take anything on I usually like to continue with it you know but I think the class, it is you know to encourage one another don’t you with a group' (ID19 Female 70-74 years FAME)*
	Lack of time	*‘My main difficulty is that life gets in the way because we have quite a busy life’ (ID4 Female 70-74 years FAME)*
		*‘I am a busy person, I do try and fit in as much as I can and as I say I paint and it annoys me when I haven’t got enough time to do my painting so you know I like to put some time aside to paint’ (ID8 Female 75-79 years FAME)*
		*‘I just think I am not that sort of person, I have got enough to do without being you know… taking exercise up. In a while I will have, I have got all of my planting to do, all my pots and things so I don’t think anything would encourage me to go to the gym or anything’ (ID28 Female 75-79 years OTAGO)*
		*‘would have looked to join a class with people, there is a class that is not too far from where I live and I have looked at that once or twice and thought about going but circumstances are that I just haven’t got the time.’ (ID1 Female 70-74 years OTAGO)*
	Mental health	*‘I can’t go any further and my legs just seize up and I feel mentally tired as well as physically tired’ (ID30 Female 75-79 years OTAGO)*
		*‘I think you can quite easily get depressed when you’re really tired so you have to sort of shake your feathers and say oh come on get on with it’ (ID6 Female 70-74 years FAME)*
Environmental	Convenience	*‘You don’t always want to be going down town’ (ID2 Female 80-84 years OTAGO)*
	Follow on activity	*‘It needs a follow up really if you are wanting to help people getting older because that… it is like taking a step forward and then you just slip back, human nature’(ID18 Male 75-79 years FAME)*
	Technology	*‘Well if you have not used technology in your working life in your life, you’re going to find it very difficult to use technology, nowadays aren’t you?’ (ID29 Female 70-74 years OTAGO)*
	Financial	*‘it does make you go because you have paid for it in advance’ (ID11 Male 70-74 years FAME)*
		*‘I mean when you have got a pension you do struggle to pay for such as gyms and things like that so if I could find something that was reasonable then I would probably do more’ (ID30 Female 75-79 years OTAGO)*
		*‘yes you need transport, which I have got……… I have got a free bus pass and the buses are only there across the road’ (ID28 Female 75-79 years OTAGO)*
	Accessible Transport	*‘yes and physically I wasn’t driving then so I couldn’t get to where they were’ (ID10 Female 85-89 years FAME)*
	Weather	*‘Yes I think the weather does, I think you know if it was pouring down with rain or something like that, I would probably be reluctant to go out and walk far in it.’ (ID11 Male 70-74 years FAME)*
		*‘Erm… the weather, you know like I was saying if it is not a very nice night you obviously don’t go or if it is cold or snow’ (ID1 Female 70-74 years OTAGO)*
	Safety	*‘Well you read the media of people being attacked, today there is someone being attacked and phone taken off her, that sort of thing’ (ID29 Female 70-74 years OTAGO)*
		*‘As long as it is sort of day time and you’re not travelling too far’ (ID6 Female 70-74 years FAME)*

### Physical facilitators and barriers

Maintenance of PA resulted in improved physical autonomy. Participants were more likely to engage in and maintain PA when they positively evaluated its benefits. The physical benefits reported were similar in both FaME and OTAGO programmes and included improvements in suppleness, balance, mobility, strength and confidence related to a reduction in falls. These represented core intrinsic rewards from taking part in ProAct65+ and helped to facilitate maintenance.

Deterioration in physical health was identified as a barrier to continuation of PA in both groups, this included developing joint problems such as arthritis. Suffering with a cough or cold, lung problems or side effects of prescribed medication were other commonly quoted barriers to maintaining PA.

Reports of witnessing physical deterioration in partners or friends promoted maintenance.

### Social facilitators and barriers

Social interaction emerged as a prominent theme facilitating maintenance of PA amongst FaME participants. The development of friendships in the class setting was repeatedly quoted as an advantage of the class. These friendships were maintained by joining new PA classes with their newly formed friends after the end of the programme, improving participants’ social support network.

Enjoyment of the exercise programmes was identified as a key reward promoting feelings of motivation and continuation of PA. Motivating factors specific to FaME included the benefit of having to leave the house for organised structured activity. Participants felt they would find distractions within the home environment reducing their commitment to doing the home-based exercises. In addition, motivation was gained by exercising alongside others.

Participants of OTAGO reported they were more likely to maintain PA levels if they lived with a partner, through motivating and encouraging one another. For both groups, positive feedback and evaluation of PA from friends and family contributed to the participants’ perception of “normal” levels of PA, influencing subjective norms and maintenance of PA. Maintenance was mainly seen in those who were previously in the habit of exercising before the programme.

Those in caring roles felt this activity made them more physically active, however it also acted as a barrier preventing participation in structured organised PA.

Barriers identified were similar in both groups, which included lack of time and having a ‘busy life’, suggesting PA was not a priority. Different reasons were given by participants for a lack of PA, highlighting competing demands on their time. These included attending clubs or social activities which participants felt were beneficial for keeping their minds active and socialising; doing volunteer work or hobbies such as painting, shopping, baking, reading and knitting. A greater emphasis was placed on these activities compared to PA.

### Psychological facilitators and barriers

‘A lot of it is motivation’ was an important theme identified in PA maintenance. Sources of motivation arose from the perceived convenience, measurable activity, organised structured activity and use of technology.

Measurable activity with pedometers and tick charts were discussed as external motivating factors by participants in both groups. These allowed participants to recognise improvements in their PA levels encouraging and facilitating maintenance. Self-efficacy and development of a positive routine or habit was perceived important in the OTAGO group to maintain PA levels.

There was evidence of health beliefs which discouraged maintenance of PA, these included thoughts of an inevitability of the ageing process ensuing once PA was discontinued and worsening of joint pains secondary to participating in PA.

Mental health and participants’ attitudes towards exercise emerged as important themes. Participants felt their attitude or that of others to old age may have a negative impact on PA maintenance. Depression or low mood was a barrier to PA maintenance affecting motivation, resulting in fatigue and higher levels of social isolation. Memory impairment was identified as another important barrier to maintenance.

### Environmental facilitators and barriers

Location and availability of exercise programmes to move onto once FaME or OTAGO had ended were important in maintaining PA. These included proximity to venues offering a range of different types of PA and exercise classes geared specifically towards older adults. When classes already existed, participants sometimes felt lack of advertisement created a barrier to making them aware of them.

Technology provided a source of motivation and encouragement through external feedback. Across both groups individuals reported they used apps on smartphones or pedometers to help maintain PA. Some participants felt it would be difficult to start using technology as they lacked prior experience and cost emerged as a prohibitive factor to using technology.

Transport was considered either a facilitator or the lack of it as a barrier for maintenance. Those that had their own car, commented location of facilities was unimportant. Easy access to a car park increased the likelihood of visiting a facility. A free bus pass meant participants could travel to take part in PA.

The weather had a detrimental impact on participant’s PA levels. Wet, snowy and icy weather prevented individuals from participating in PA outside the home, due to a fear of falling. Cold weather was felt to have a negative impact on joint pain, inhibiting PA maintenance.

The timing of classes or planned PA also acted as a barrier to keeping active, with some participants reporting classes were held at inconvenient times of the day. Personal safety was a concern for those attending classes running late into the evening, more important when the days were shorter in the winter months. Conscious efforts were made to time walks during daylight hours. Some individuals felt uneven terrain or hard surfaces stressed their joints acting as a barrier to maintain PA.

Cost or affordability was considered both a facilitator and a barrier. Some participants reported they found it expensive to access local facilities where they had to sign a contract and pay an initial set fee, particularly due to being on a fixed income with a pension. Whereas for others the initial upfront fee, if affordable, promoted maintenance by encouraging a commitment to attending the classes they had already paid for.

## Discussion

### Main findings

The results of our study show that PA maintenance behaviours in older people are complex with multiple influences. Our findings add to the body of literature for older adults, which is often a neglected group and discussed in the context of well accepted behaviour change theories. Our results showed perceived improvements in physical health, social support and interaction, exercising alongside others, encouragement from friends and family and positive visual feedback all acted as facilitators to PA maintenance. Creating routines and making PA habitual were important for OTAGO participants, whilst making a commitment to attend exercises classes was important for FaME participants. Witnessing a physical deterioration in others, physical or mental health problems, competing interests, poor weather, safety fears, lack of transport and location of facilities all acted as barriers to PA maintenance. Cost was both a facilitator and a barrier to maintaining PA.

#### Strengths and limitations

This was a qualitative study of people that participated in a large, randomised controlled trial of usual care versus home and group-based exercises to increase PA and reduce falls. Our study provides some explanations as to why strength and balance exercise programmes work to increase physical activity.

Strengths of this study include the large number of interviews conducted allowing thematic saturation, and coding by two researchers. Further coding of some transcripts by a PPI member provided a lay perspective on themes emerging. Interviewer shadowing by both researchers conducting the interviews helped ensure consistency in technique.

This study was subject to certain limitations. Whilst 54% of those invited to the study expressed interest in participating, deaths and illnesses would have affected who the GP sent invitations to. Those not invited, those not expressing interest, or those expressing interest who were not interviewed, may have differed from those who were interviewed with regards to maintenance of physical activity. We did not have ethical approval to approach those not responding to study invitations, so we were unable to explore their characteristics and reasons for non-response. Interviews were conducted between 4 and 6 years after the end of the ProoAct65+ exercise programme, participants may have recalled facilitators and barriers to PA maintenance more easily if interviews had occurred closer to the end of the exercise programme. However, the findings are similar in content to what is already known about barriers and facilitators to PA maintenance, which might suggest that delayed recall does not alter themes. For individuals who viewed participation in the ProAct65+ trial as a significant life event, as taking part was demanding and intense (in terms of the volume of data collected). And if life was positively changed through the social interaction provided by the FaME programme or increased independence due to improved activities of daily living, then recall could be valid for an extended period of time [45].Social desirability bias may have occurred, as participants may have over reported PA levels and selection bias may have occurred if those who were more PA were more likely to participate. In addition, fewer participants were men and only one participant belonged to a Black & Minority Ethnic (BME) group, so their views are not well represented in our study.

#### Comparisons with existing literature

Several other studies have shown that good physical health status [46–50] and being physically fit [51–53] encourages PA maintenance. These are consistent with our study findings where poor or deteriorating physical health acted as a barrier to PA maintenance.

Our study found those living with a partner reported it supportive for the maintenance of PA. The literature shows a positive association with PA maintenance in those who are married [46, 48, 54]. However, evidence for social support promoting PA maintenance is limited [50, 53, 55]. Perceived social support received from exercise instructors [56], walking companions, friends and sports exercise group (e.g. volleyball, basketball, aerobics and badminton) members [32, 53] has been found to be positively associated with PA maintenance.

Positive mood has been associated with PA maintenance [49, 51], and in our study participants reported depression and low mood acting as a barrier. Feelings of safety within the neighbourhood and perceived accessibility of facilities has been positively associated with PA maintenance [57], similar to our study findings.

PA maintenance is problematic, identification and syntheses of existing theory may help us to better understand PA maintenance in older adults, which can be used to inform future intervention development. Our findings are consistent with several theories of behaviour change, although no single theory encompasses all our findings. PA maintenance was facilitated by positive evaluation of its benefits consistent with the theory of planned behaviour (TPB) and regulation theory (RT) [58]. Remaining independent and improving confidence in balance helped maintain autonomy, consistent with the self-determination theory (SDT) [59, 60]. Perceived susceptibility in terms of the risks individuals faced by not taking part in PA played a role in maintenance; consistent with the health belief model [61]. In contrast, some older people perceived physical deterioration in older age to be inevitable or outside of their control demonstrating a lack of self-efficacy acting as a barrier to maintaining PA, consistent with social cognitive theory (SCT) [62–64] and consistent with other studies self-efficacy findings [46, 47, 50, 53].

Structured organised activity provided greater motivation than home based activities, which may be undertaken alone. In older age, social activities and inclusion become increasingly important due to death of partners and friends, deterioration in health and lack of mobility which can result in social isolation. In our study social interaction featured as a prominent theme for the maintenance of PA. The importance of the social context and environment in promoting behaviour change are well described by the SDT, organismic integration [59] and cognitive evaluation theories (CET) [65]. FaME classes provided this ideal environment, contributing to participant’s enjoyment of the classes further supported with peer motivation during classes, resulting in maintenance behaviour consistent with the RT. [66] In addition, positive feedback from peers, family and friends, from having their progress monitored by completing exercise diaries or from using technology such as pedometers facilitated maintenance by reinforcing subjective norms consistent with the CET and TPB [67]. A study [50] following up participants of the PACE-UP trial [68] where pedometers were used to increase walking in adults aged 45–74 years, showed pedometers helped with ‘kick-starting’ activity and subsequently supported maintenance of PA.

Those in the OTAGO group required higher levels of self-efficacy to maintain PA, this is explained through the social cognitive theory (SCT) [64] and health belief model due to the lack of social support, but had more control (TPB, SDT) over when and how much PA was completed. Where there was failure in development of a routine consistent with the Habit Theory (HT) [69, 70] this was often due to lack of prioritisation of PA, conversely development of this contributed to maintenance.

## Conclusion

We have identified a range of factors which, if incorporated into community-based exercise programmes should facilitate exercise maintenance and help commissioners when designing exercise programmes for older adults. To meet the varied needs of older adults and to encourage maintenance of increased levels of PA once exiting local community based exercise programmes, a range of physical activity programme options should ideally be available. This will help older adults choose what, why, when and how to maintain their increased PA. Providing monitoring and feedback [71, 72] and increasing self-efficacy related to PA are important to PA maintenance.

Evidence has shown that social interaction promotes PA maintenance. This could be implemented through ‘buddying schemes’ with support from group members or exercise partners [33, 53] which has been shown to be positively associated with maintenance. Alternatively, the provision of subsidised structured PA classes participants can continue onto may promote maintenance. Signposting to relevant facilities and classes after exercise programmes have ended, has been identified as being important to help facilitate maintenance of PA. The option of organised community transport to PA facilities would address the transport issue for those who report difficulty accessing facilities. For those preferring home-based exercise, ongoing monitoring and peer meetings would support maintenance.

## Supplementary information

**Additional file 1.** Keeping Active Interview Guide.

## Data Availability

The datasets generated and analysed during the current study are not publicly available as permission was not sought from participants to share interview transcripts.

## Abbreviations

CET: Cognitive evaluation theories
HT: Habit theory
PA: Physical activity
PPI: Patient and public involvement
ProAct 65+ trial: Group (FaME) and home based (OTAGO) exercises
RT: Regulation theory
SCT: Social cognitive theory
SDT: Self-determination theory
TPB: Theory of planned behaviour
