# Supplementary material for: Use it or lose it: a qualitative study of the maintenance of physical activity in older adults
Source: BMC Geriatr. 2019 Dec 12;19:349. doi: 10.1186/s12877-019-1366-x (PMC6909612; doi:10.1186/s12877-019-1366-x)
Supplement: Supplementary file 1 — Additional file 1. Keeping Active Interview Guide. [file 12877_2019_1366_MOESM1_ESM.docx]

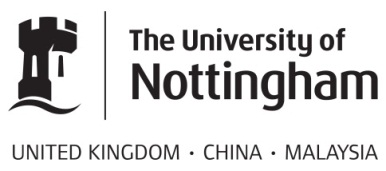


**Additional file 1: Keeping Active Interview Guide**

**(ProAct65+ participants)**

**Manuscript: ‘Use it, or lose it’: maintenance of physical activity post participation in a strength and balance training programme for older adults. A qualitative study.**

Thank you for agreeing to take part in this study. We are interested in finding out more about what you do to keep active and about the exercise classes or home exercises you did as part of the ProAct study. In the interview I will ask you about physical activity; by this I mean any movement of your body which requires you to use energy. This includes exercise you might do, for example, swimming or walking to for fitness, but it also includes other types of activity such as gardening, housework, dancing, DIY, walking to the shops etc. If you are not sure what any of the questions mean, please let me know. At the end I will ask you if there is anything else you would like to add that hasn’t been covered by the questions I’m going to ask.

The interview is going to cover three different time periods. First we will start by talking about the time when the ProAct65+ trial started, then we will talk about the time period immediately after the ProAct65+ trial finished. Then finally we will talk about now.

1. Just so we have this on the tape recording of the interview, can you tell me whether you were in the exercise classes or the home exercise programme group as part of the ProAct65+ trial?
2. Could tell me a little bit about your thoughts about exercise classes/home exercise programmes?
   1. If you think back to the time before the ProAct trial started, how physically active would you say you were before the ProAct65+ study?
      1. What kinds of physical activity did you do and how often?
   2. Had you been to any exercise classes/taken part in any home exercise programmes before the proAct65+ study?
      1. If yes, how long before the ProAct65+ study was this?
      2. How did your experience of those classes/home exercise programme affect your views about taking part in the ProAct 65+ classes or home exercise programme?
   3. Can you tell me how you felt when you found out you were in the exercise class/home exercise group in the ProAct65+ study?
      1. Were you looking forward to anything in particular about the classes/home exercise programme?
      2. Was there anything about the classes/exercise programme that you were concerned or unsure about?
      3. How do you think you would have felt if you had been in the other exercise group (i.e. home exercise programme or exercise classes).
   4. Thinking now about the classes/home exercise programme in the ProAct65+ study, how would you describe your feelings about them?
      1. Were they like you expected them to be?
      2. Would you have changed anything about them if you could?
      3. Would you join another similar class/home exercise programme in the future?
      4. Do you think the classes/home exercise programmes benefitted you in any way?
         1. How did the classes/home exercise programme affect how physically active you were?
         2. Were there any other benefits?
            1. If so, which were most important for you?

So now, if you think about the end of the ProAct65+ trial and what you were doing in the first couple of months after the exercise/home exercise program ended

1. Can we discuss what happened when the classes/home exercise programme came to an end?
   1. How did you feel when the ProAct65+ exercise classes/home exercise programme were/was coming to an end?
   2. What happened to your physical activity straight after the end of the exercise classes/home exercise programme?
   3. What kinds of physical activity were you doing in the first few months after the exercise/home exercise program ended and how often?
      1. Why did you choose those types of physical activity as opposed to other types of physical activity?
   4. What things affected how physically active you were in the first few months after the classes/home exercise programme ended? (hand participant cue card and ask the participant to refer to the cues to help answer the question)
      1. Your health or that of your partner?
      2. Any pain experienced whilst being physically active?
      3. Any falls you might have had or concern about falling? The fear that physical activity might worsen rather than improve other conditions (e.g. arthritis, respiratory disease, or symptoms like angina for example)
      4. The social benefits of the activity
      5. Views of family and friends?
      6. Availability of opportunities to be physically active? (give suggestions e.g. places to walk, swimming pools, leisure centres, clubs such as bowls, tennis etc, group activities such as walking or cycling groups, exercise classes e.g. yoga, tai chi, aquarobics etc, dancing, gardening)
      7. Help to be physically active (e.g. transport to places to do exercise, someone to go with, etc)
      8. The cost of being physically active?
      9. Safety of your neighbourhood?
      10. Weather?
      11. Other factors – e.g. any other benefits or risks that might influence physical activity?
   5. What do you think would be the best way of encouraging people like you to carry on being physically active at the end of classes/home exercise programmes?

Now I am going to ask you about what you are currently doing.

1. It’s now several years after the classes/home exercise programmes have ended.
   1. How physically active are you now?
      1. What kinds of physical activity do you do now and how often?
      2. Why do you do these types of physical activity as opposed to other types?
   2. What kinds of things affect how much physical activity you do nowadays? (ask participant to refer to cue card given for the previous question. Again, to help answer the question)
      1. Your health or that of your partner?
      2. Any pain experienced whilst being physically active?
      3. Any falls you might have had or concern about falling?
      4. The fear that physical activity might worsen rather than improve other conditions (e.g. arthritis, respiratory disease, or symptoms like angina for example)
      5. The social benefits of the activity
      6. Views of family and friends?
      7. Availability of opportunities to be physically active locally? (give suggestions eg. places to walk, swimming pools, leisure centres, clubs such as bowls, tennis etc, group activities such as walking or cycling groups, exercise classes e.g. yoga, tai chi, aquarobics etc, dancing, gardening)
      8. Help to do exercise (e.g. transport to places to do exercise, someone to go with, etc)
      9. The cost of exercise?
      10. Safety of your neighbourhood?
      11. Weather?
      12. Other factors? – e.g. any other benefits or risks that might influence physical activity?
2. How do you feel about using technology like pedometers, mobile phone apps, exercise videos/DVDs or exercise games to help you keep physically active?
   1. Have you ever used technology to help keep yourself physically active?
   2. Would you ever consider using anything like this in the future?
      1. If yes, what might encourage you to use it?
      2. If no, why not? What would prevent this/put you off?
3. If you wanted to increase the physical activity you currently do, how might you go about this?
   1. What would help you to be more physically active?
   2. What would make it harder for you to be more physically active?
   3. Is there anything else about the Proact65+ classes/home exercise programme or about physical activity/activities that you would like to tell us?
4. We know from previous studies that what people close to us, like partners or family members think about physical activity influences how active we are. As mentioned in the study information leaflet we are interested in talking to partners/carers/close friends of people we have interviewed to find out their views about physical activity.
   1. Is there someone close to you that you would be happy for me to contact to see if they would be happy to be interviewed, either face-face or by phone as part of our study?
   2. If yes, please can you ask them if they would be happy for you to give me their contact details so I can contact them?
   3. Would you be happy for me to phone you next week to see if they have agreed?

That’s all my questions now, so thank you very much for taking part in the interview. What you’ve told me will be very helpful to our study.
